# Pollen-foraging preferences of honey bee colonies (*Apis mellifera* L.) based on plant bloom timing in an urbanized Northeast U.S. habitat

**DOI:** 10.1371/journal.pone.0335828

**Published:** 2025-10-30

**Authors:** Cole F.P. Moran-Bariso, David C. Gilley

**Affiliations:** Department of Biology, William Paterson University of New Jersey, Wayne New Jersey, United States of America; Oda Bultum University, ETHIOPIA

## Abstract

To successfully provision the colony with protein and other essential nutrients, honey bee colonies track ephemeral floral food sources and make adaptive group decisions about which pollen species to reject, which to collect, and in what quantities. This descriptive study has two objectives; 1) to document pollen diet-choice decisions (both acceptance and rejection) in freely foraging honey bee colonies at high temporal resolution over an entire foraging season, and 2) to create a phenological calendar for the mutualism between honey bees and their pollen-food source plants that can be used to assess possible future phenological mismatch. We used pollen traps to harvest pollen weekly from two honey bee colonies from May until October (2023) to determine the relative abundances of pollen sources utilized. We simultaneously monitored in the field plant-bloom timeframes of 41 known honey bee pollen-source plants within the colonies’ foraging range. Honey bees collected at least 33 plant species in detectable amounts, with an average of 6 species in any single 24-hour colony harvest. An average of 13.7 known food sources were in bloom per week, with foragers utilizing 5.6 sources on average per week, showing that rejection of food sources is a major element of pollen-diet choice. The identity of accepted and rejected sources fluctuated every week. Foragers adopted novel pollen source species constantly throughout the season, on average 2.3 new sources per week, suggesting effective tracking of ephemeral floral sources which was intertwined with reliance on a set of abundant perennial herbs. These data provide a unique high-resolution picture of adaptive group foraging decisions in honey bees over an entire foraging season. Pollinator diet-choice dynamics are increasingly important to understand because they not only drive angiosperm reproduction in both natural and agricultural ecosystems but also are expected to be impacted strongly by climate change.

## Introduction

Pollen foraging is a critical link in the mutualism between honey bees and flowering plants which is vital for both agricultural and natural ecosystems. Collection of pollen from diverse plant sources by honey bees has been well documented in recent years by studies across the globe in a diversity of habitats. The rationales for these studies include design of pollinator-friendly urban landscapes [1–3], documentation of floral resource variation in agricultural ecosystems [4–6], informing agricultural practices to support pollinators [7–10], conservation of bee floral habitats [11,12], comparison of species-specific foraging strategies [13], and investigation of connections between diet diversity and both immunocompetence [14,15] and pesticide resistance [16]. Common to all of these studies is the demonstration of significant seasonal variation in the spectrum of pollen species collected by honey bee colonies. Much of this seasonal variation in pollen harvest composition is no doubt attributable to the ephemeral nature of floral food sources. However, the adaptive fine-tuning of social foraging in honey bees (exemplified by [17]) suggests that temporal variation in pollen collection is best understood as the outcome of optimal foraging strategies against the background of a complex and changing floral environment.

Colony pollen-foraging choices over the course of a season are expected to be adaptively tuned to colony lifecycle events. In the early spring, colonies prioritize reproductive efforts by focusing on brood rearing and reproductive swarming opportunities, which generally peak between mid-May through late June in our Northeastern U.S. Region [18,19]. During this early-season period rapid brood production translates directly into reproductive success through colony fission [20]. Therefore high abundance, diversity, and nutritional content of pollen, which support brood development and impact adult health [14,21–23, reviewed by 24], are of critical importance. For example, supplementing colonies with pollen in the spring has been shown to increase brood-rearing [25] and pollen stressing spring larvae has been shown to produce adult workers that are poor foragers and waggle-dancers [26]. In the middle season, established colonies shift from growth to maintenance of the worker population. During this period, colonies require uninterrupted pollen supply to sustain worker populations, ongoing brood rearing, and recovery from swarming population loss. Honey bee colonies have been shown to precisely regulate pollen foraging efforts in response to both supply and demand [27–29]. In the late season, colonies shift from maintenance to winter preparation, as they gather and conserve resources vital for successful overwintering survival. Honey bee colonies are known to store a reserve of pollen of approximately 75g from fall foraging throughout the winter [30–32]. Colonies with large, diverse winter pollen stores may gain a reproductive advantage in the next active season by using these stores for brood production before the main spring bloom period. Limited late-season pollen diversity as well as abundance could result in the storage of nutritionally suboptimal pollen, reducing its capacity to sustain colony health through winter and support the development of high-quality spring brood [23]. Indeed, limiting amounts of fall pollen stores have been shown to affect spring worker survival [32].

Colony pollen-foraging choices on daily time scales are expected to correspond with predictions from optimal diet models where nutritional intake is maximized [33]. Pollen food-choice decisions are expected to be based on encounter rates, handling times, and nutritional values of each available pollen source species. Encounter rates and handling times will be determined by proximity of plants to the nest, flower density, patch size, growth habit (e.g., trees, herbs, vines), and flower morphology. The nutritional value of the pollen to honey bees includes total protein and lipid mass as well as amounts of specific nutrients such as vitamins, minerals, or essential amino acids (reviewed by [24,34]). Nutritional variation among floral sources utilized by honey bees has been documented for total protein fraction [21,35] and amino acid compositions [36]. These variations in nutritional content have been shown to affect pollen foraging preferences [21,35,36], but the relative importance of each nutritional component for foraging preferences remains unclear. Freely foraging honey bee colonies should be expected to accept certain pollen sources and reject others, and regularly shift these preferences as new sources become available while old sources fade.

Honey bee pollen foraging is expected to be highly dynamic, as colonies track potential food patches as they shift both spatially and temporally. These dynamics are a challenge to document due to the need for sampling at a high temporal resolution over an extended period of time and the large land area covered by a single colony’s foragers. Additionally, and important for the rationale of these present study, is that understanding true foraging preferences requires documentation of both the collected *and rejected* food sources. We consider understanding these dynamics a high priority given that these foraging decisions lie at the very heart of the services honey bee colonies provide to both natural and agricultural ecosystems. The purpose of the present descriptive study is to examine pollen food-acceptance and food-rejection dynamics in freely foraging honey bee colonies in a heterogenous urbanized environment over the course of an entire foraging season.

Understanding the pollen food preferences of generalist pollinators is increasingly important in the face of climate change. Climate variables such as temperature and precipitation will affect food plant phenology including pollinator-relevant events such as first, peak, and last bloom dates [37–39]. Generalist pollinators such as honey bees are likely to react to phenological changes in their food plants by altering patterns of acceptance and rejection of particular food sources as a season progresses. Once-adaptive food-choice decisions may become increasingly maladaptive as the seasonal cycle of events within a honey bee colony becomes increasingly mismatched to the phenology of their key pollen-source plants. Such phenological mismatch has been cited as a causal factor behind worldwide pollinator decline (reviewed by [40]). To be able to detect such phenological mismatch between pollinators and flowering plants and to evaluate its extent, simultaneous phenological calendars are needed for both food plants and pollinator foraging behavior. Such phenological calendars would ideally have high temporal resolution, cover the entire foraging season in one location, and be integrated with data on local heat accumulation. Creating such a phenological calendar for the mutualism between honey bees and their pollen-food source plants is a secondary goal and an important part of the rationale for the present study.

Our approach for addressing both the primary and secondary goals above was to collect pollen weekly from two honey bee colonies in a suburban location over the course of an entire foraging season (May until October) while simultaneously monitoring the bloom timing of known honey bee pollen source plants within the colonies’ foraging range.

## Methods

### Apiary and bee colonies

Two honey bee colonies were established on 14 April 2023 at a rooftop apiary at William Paterson University in northern New Jersey, USA (apiary GPS coordinates: 40.9476195, −74.1967779). Each colony was transferred from a nucleus box into two ten-frame hive bodies on 20 April. Pollen traps and an additional empty hive body were added to each colony on 11 May. Colonies were managed with minimal invasiveness with the goal of preventing loss due to swarming while restricting their size to three hive bodies to minimize colony variation and allow for accurate surveys of their condition. For Colony 1, five full honey frames were removed and replaced with empty frames on 6 June, and 10 frames removed and replaced on 28 June. For Colony 2, five full honey frames and five brood frames were removed and replaced with empty frames on 23 May to prevent swarming. Visual surveys of brood frames, honey frames, empty frames, adult bees, and presence of queen cells were conducted on 22 May, 5 June, 21 June, 12 July, and 26 July. During these hive surveys, visual observations were made to estimate the frame equivalents of brood and adult workers, and the status of the queen was noted for both colonies. The weight of the colonies was also tracked on a weekly basis over the 20-week season using Bluetooth hive scales (Broodminder.com). Overall, the two colonies were similar, with small differences between their brood and worker populations; Colony 1 had an average of 7.5 brood frames (1.94 SD, 0.87 SEM) and 15.86 worker frames (SD 2.74, SEM 1.23), while Colony 2 possessed 10.4 brood frames (SD 4.28, SEM 1.91), and 12.6 worker frames (SD 3.45, SEM 1.54). The weekly hive weights were also similar between the two colonies throughout the 20-week season with Colony 1 possessing an average weight of 85.75 kg and Colony 2 possessing an average weight of 80.88 kg.

### Pollen collection

24-Hour pollen samples were collected from the two colonies using Sundance pollen traps (Northeast Center for Beekeeping, LLC). Pollen traps with removable drawers and an adjustable entrance which could be toggled open or closed were placed underneath the lowest hive body of each honey bee colony. Pollen traps were opened on the first weather-permitting day once a week for a 24-hour period at the same time each week (8:45am + /- 30 minutes) and closed following the 24-hour period. Weather-permitting days were defined as days where the 24-hour forecast predicted no weather that would interfere with pollen foraging such as rain, thunderstorms, or cold temperatures (less than 55°F). This criterion was established to ensure that pollen harvests did not misrepresent floral sources that were attractive to bees for only part of the day. When the trap was in the open position, bees entering were forced to travel through a screened maze-like matrix which removed from their corbiculae pollen they were bringing back to the colony. The pollen pellets removed from their corbiculae fell into a lower chamber with a drawer the bees were unable to access. Pollen from each colony drawer was collected in separate 50mL centrifuge tubes after closing the traps. These pollen samples were weighed without drying prior to being stored in the freezer at −20°C.

### Pollen identification and quantification

Subsamples of 200 randomly selected pollen pellets were used to determine plant identities. This amount, approximately 1.5 g, is similar to other recent studies [1,6,41] and consistent with the findings of [42]. Each subsample was suspended in a 20% glycerin solution at a uniform dilution that prevented overlap among pollen grains when viewed at 400x. Samples were homogenized using a vortex mixer on high power for three minutes. 10μl of each diluted homogenized sample was pipetted into one well of an Improved Neubauer Brightline hemocytometer (Hausser Scientific) using a P-20 micropipette. Compound light microscopy at 400x magnification was used to identify source plants to the lowest taxonomic level possible based on size, color, texture, and morphology, consistent with the methods of [1,12,21]. Pollen identifications were cross referenced with online pollen databases including Paldat.org and the Cornell University Pollen Grains Reference Library to ensure accurate identification of each pollen grain. Knowledge of local flora, observations of honey bee foraging, and pollen harvest coloration were also used to narrow down the possible identities of pollen grains. Visually distinguishable pollen grains that could not be attributed to a plant taxon are referred to hereafter as taxon-unattributed and assigned a unique pollen identification code (e.g., “Taxon-Unattributed A”) following the methods of [12]. The relative abundance of each pollen source was calculated as the number of grains of that type in the hemocytometer well divided by the total number of pollen grains in the hemocytometer well. Relative abundance counts were conducted by counting all pollen grains within the nine boxes of an x-pattern of the main 5x5 center hemocytometer grid. The nine box counts were summed for each of the two wells, then the two well counts were averaged for each colony for each sample date. Two counts were conducted for each weekly sample from each colony, totaling 80 counts over the course of the entire season. The total number of grains for the two counts differed by an average of 13.9%. All raw data in the form of pollen counts are provided as supplementary information with this paper (S1 Table). On average, 140 unique grains were counted per well for a total of 280 grains on average per pollen sample. This pollen identification and quantification process was used to examine a total of 13,980 pollen grains. Of the 13,980 pollen grains examined, 9,835 were quantified and attributed to a plant taxon, 2,572 were quantified but taxon-unattributed, and 1,573 could not be attributed to a plant taxon or quantified due to unusual morphologies and low relative abundances. These 1,573 grains could not informatively be included in the analyses of this study resulting in conservative (i.e., minimum) estimates of diet richness and diversity. Taxon-unattributed grains were included in all analyses except the phenological calendar (Fig 5) as they represented distinct, but unidentified, species.

**Fig 1:**
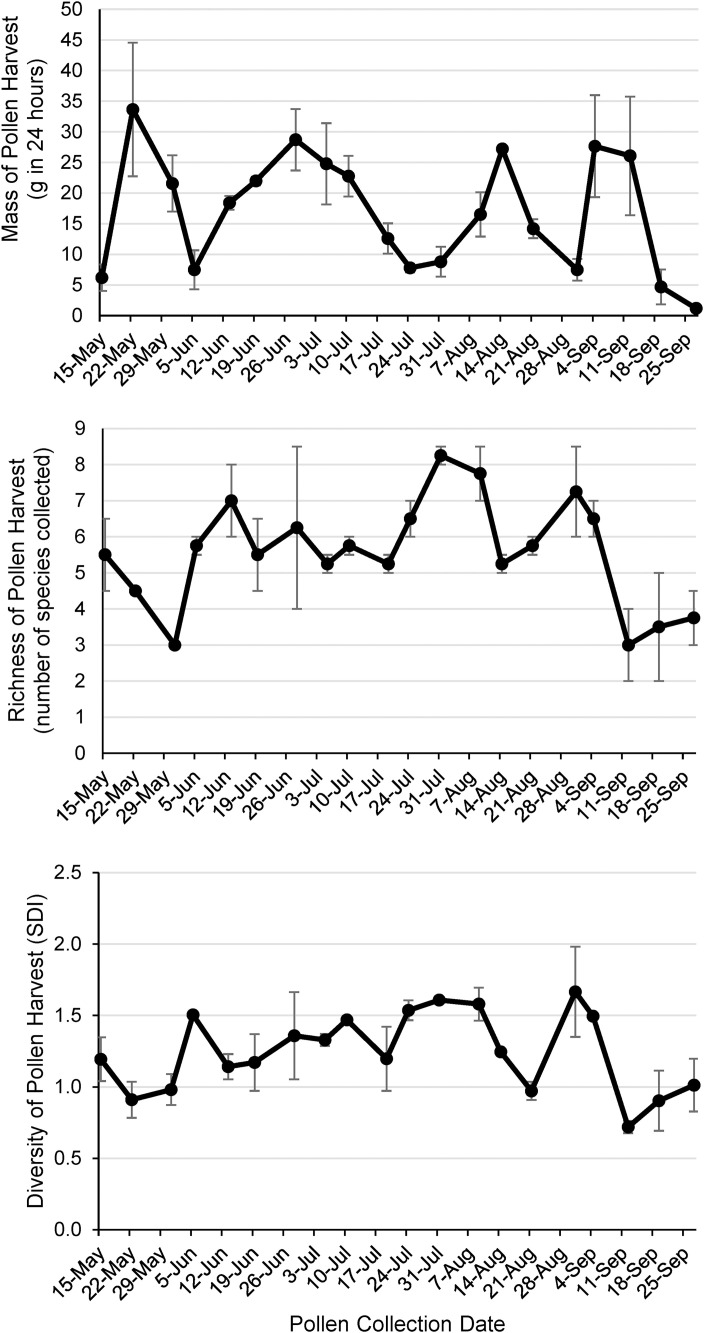
Description of the total pollen harvest from two honey bee colonies over the 2023 foraging season. Panel A: average wet mass of pollen harvest, Panel B: average species richness of pollen harvest, Panel C: average Shannon diversity index of pollen harvest. Pollen harvests were weekly 24-hour samples of collected pollen from two honey bee colonies. Taxon-unattributed but visually distinguishable pollen grains are included in this index. Error bars represent the standard error of the colony means.

**Fig 2:**
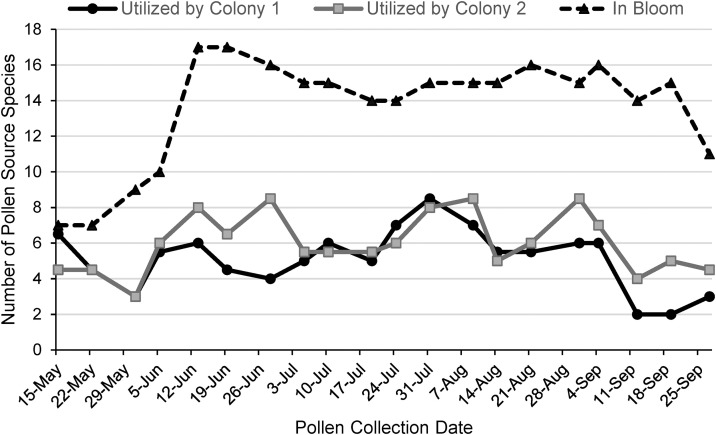
Comparison of the number of pollen source species utilized to the number available. The number of pollen source species utilized by two honey bee colonies throughout the 2023 foraging season are represented by solid lines. The total number of species known from field observations to be in bloom at each time point is represented by the dashed line.

**Fig 3:**
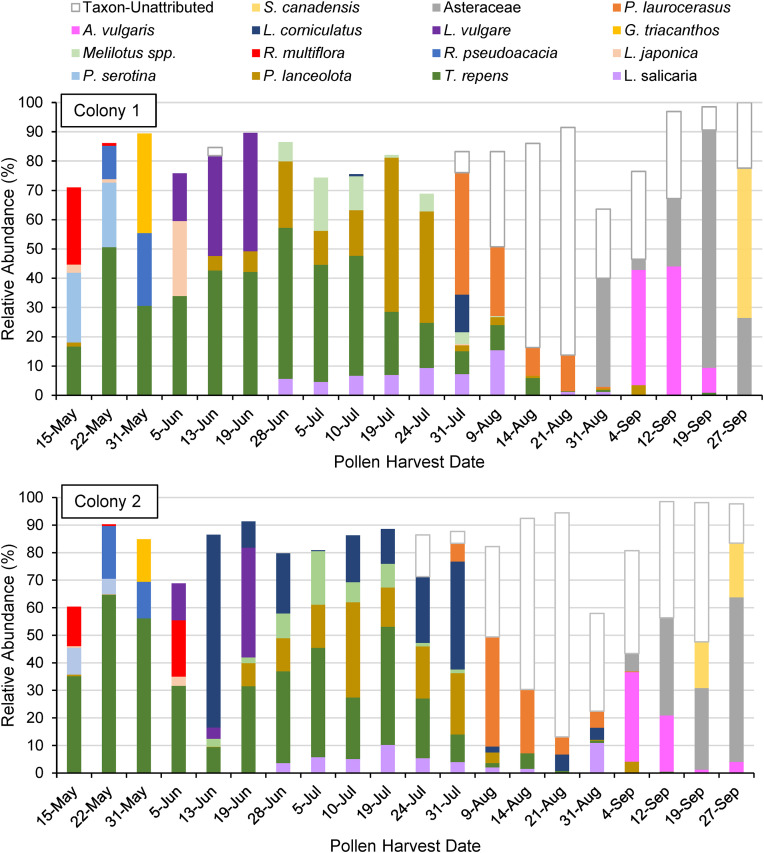
Composition of weekly pollen harvests throughout the 2023 foraging season for two honey bee colonies. Only major sources are shown for clarity; pollen source species with peak relative abundances of less than 10% are not shown (but are included in Table 1).

**Fig 4:**
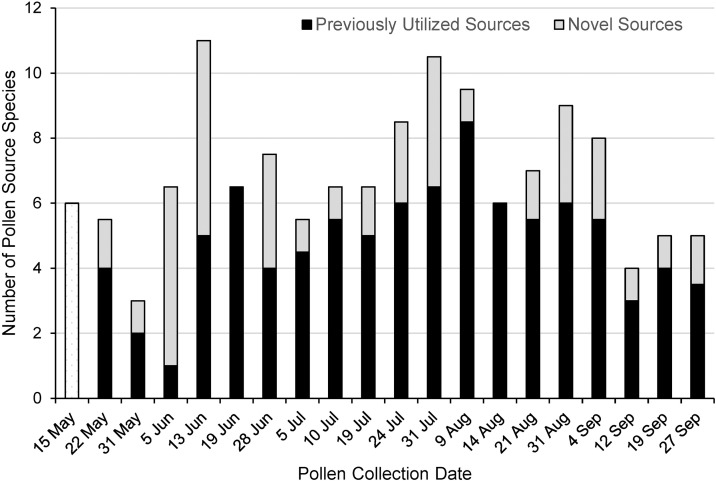
Comparison of novel versus previously utilized pollen food-source species throughout the 2023 foraging season. Data from two colonies was pooled. Includes taxon-unattributed but visually distinguishable pollen grains. Proportions are not shown for May 15 because utilization of sources before the beginning of the experiment could not be determined.

**Fig 5:**
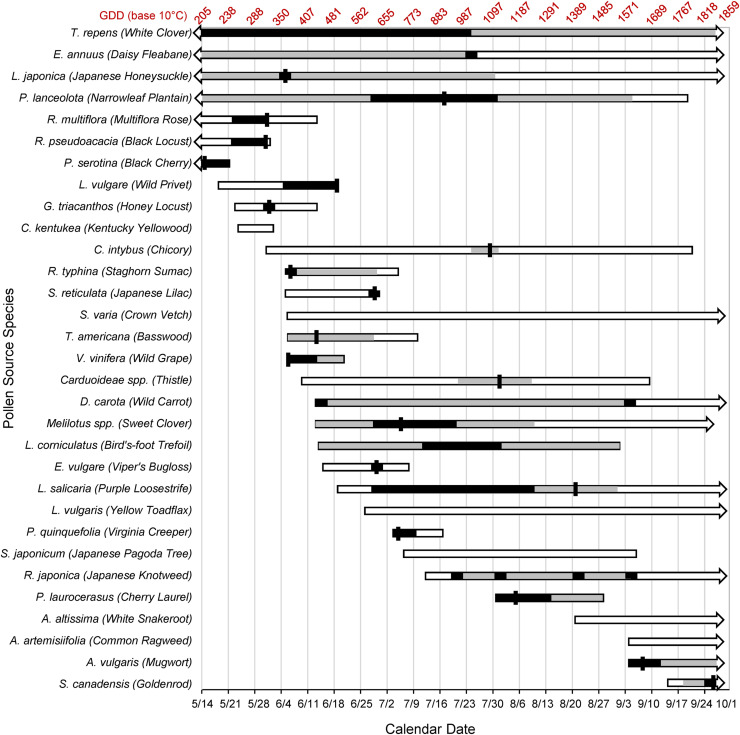
Phenological calendar of honey bee pollen foraging for 2023 season. This figure superimposes data from hive pollen traps with field observations of food-plant blooming dates. White (unfilled) bars represent the beginning and the end of observed blooming for each species. Arrows indicate possible blooming before or after the end of the experiment. Gray-filled regions indicate dates during which pollen of each species was positively identified from hive pollen traps, while black-filled regions indicate heavy collection of a particular species (above 25% of its peak relative abundance). Black vertical bars indicate the date of peak relative abundance of the species in hive pollen traps, but are only shown for species with a single distinct peak relative abundance. Pollen source species were included here if either their pollen had been observed at some point in hive pollen-trap samples, or if honey bees had been observed in the field on the plants with pollen in their corbiculae. Growing degree days (GDD, in Celsius units) are included as an alternative axis to provide a record of how the phenology relates to local heat accumulation.

### Field surveys

Field surveys were conducted twice per week over the 20-week period from 15 May – 27 September 2023, with the aim of generating a menu of available food plant sources from which the two honey bee colonies had foraged throughout the course of the entire season. The bloom timeframes of flowering plants within foraging range and known to be visited by honey bees were tracked throughout the entire season. Growing degree days (GDD) were determined using the public information tool CLIMOD2, available from the Northeast Regional Climate Center. Degree-days were calculated based on the single station closest to the location of the apiary (Calwell Essex County Airport) using the daily average-temperature method with a base temperature of 10.0°C (50.0°F), and accumulation beginning on January 1, 2023. Growing degree days are reported here in Celsius units.

Field sites were chosen based on public access within the known foraging range of the European honey bee (up to 10.9 km radius [17]), with a maximum distance of 7.02 km from the hives. Primary field sites were surveyed two to three times per week including the William Paterson University campus (40.9476195, −74.1967779), High Mountain Park Preserve (40.9519120, −74.2003221), Barbour Pond (40.9601581, −74.2267861), and Wayne Village Apartments (40.9579660, −74.2289040). Secondary sites which were surveyed less than three times per week, but at least once per week include the Wayne Hills Shopping Mall (40.9642270, −74.2387410), Everett J Faber Memorial Park (40.9620729, −74.2785748), Bridal Veil Falls (40.9512460, −74.1921299), and Laurelwood Arboretum (40.9867252, −74.2670175). Field sites were surveyed for plants which are known honey bee sources. Once a plant was found, its location was logged using GPS coordinates via Google Maps. Field observations of each plant were recorded including the presence of foraging honey bees and plant bloom status (buds forming, in-bloom, no longer blooming, fruiting). Plant blooms were considered complete when a particular plant source was no longer observed blooming in at least two different field locations. The same set of locations were surveyed throughout the entire foraging season, documenting the bloom timeframes of all previously recorded species, while noting the emergence and bloom timeframe of any new flowering plants observed in those areas.

A qualitative assessment of the relative abundance of each plant species throughout the foraging area was made following the end of the observed bloom timeframes. Ubiquitous sources present at nearly all (approximately 95%) field locations included *Trifolium repens*, *Plantago lanceolota*, *Gleditsia triacanthos*, *Robinia pseudoacacia*, *Securigera varia*, *Tilia americana*, *Styphnolobium japonicum*, and *Lotus corniculatus*. Very Common sources present in many (approximately 75%) field locations included *Lythrum salicaria*, *Chicorium intybus*, *Carduoideae spp*., *Artemisia vulgaris*, *Lonicera japonica*, *Rosa multiflora*, *Erigeron annuus*, *Prunus serotina*, *Ligustrum vulgare*, *Syringa reticulata*, *Daucus carota*, *Echium vulgare*, *Parthenocissus quinquefolia*, *Reynoutria japonica*, *Prunus laurocerasus*, *Ailanthus altissima*, and *Sambucus canadensis*. Uncommon sources present only scarcely (less than 25%) in field locations included *Linaria vulgaris*, *Rhus typhina*, *Melilotus spp.*, *Ambrosia artemisiifolia*, *Cladrastis kentukea* and *Vitis vinifera*. A Kruskal-Wallis H test [43], a non-parametric alternative to a one-way ANOVA appropriate for non-normally distributed data, was conducted to compare the total number of pollen grains collected among these three categories of plant commonness.

Land use in a 3 km radius from the hives’ location was assessed to provide further context for interpreting pollen food choices. A grid consisting of 200 m x 200 m squares was imposed on a map of this area and each square was designated as one of five land use types based on the majority percentage of land cover in each square (i.e., more than 50% total coverage). “Park” type land was defined as any green space that is not densely covered by trees, such as golf courses, or city parks with fairly open spaces; “forested” land was defined as any land with sufficient tree cover, whether it is an actual forest or a dense, relatively large outcropping of trees; “commercialized” land was defined as any public or industrial city space such as schools, company buildings, churches, etc.; “residential” land was defined as all privately-owned land such as houses and apartment complexes; and “other” represented any space without a clear means for classification, such as in the case of an equal proportion of multiple land types present. Land use was as follows: park = 4.1% of total squares, forest = 19.5%, residential = 53.0%, commercial = 15.6%, and other = 7.8%.

### Field study ethics statement

Field surveys were conducted on public land, including the High Mountain Park Preserve, or on publicly trafficked private land, upon which no permission was required to conduct this observational study. No sampling of protected plants or animals was conducted. All sampling of plants and animals was non-destructive and observational only. Honey bee colonies were managed with minimally invasive beekeeping techniques and no parts of this study required sacrifice of individuals or colonies of bees.

## Results

### Characterization of pollen-foraging choices over entire season

Honey bee colonies collected pollen from at least 33 different plant species, as identified based on unique pollen grain morphology of 12,407 quantified pollen grains from weekly samples of two colonies taken between 14 May 2023 and 27 September 2023 (Table 1). Based on these data, 23 of the 33 plant food-source species were positively identified, 2 of 33 were identified to the genus or subfamily level, and 8 of 33 could not be attributed to a plant taxon. Honey bee foragers from unidentified source colonies were observed gathering pollen from an additional 8 plant species known from previous studies as honey bee food sources, but which were not detected in the pollen samples of this study’s colonies (Table 1).

**Table 1:** A summary list of the pollen species collected by two honey bee colonies between 14 May and 27 September 2023.

Plant Species	Plant Category	Bloom Period Observed in Field	Date Range Observed in Pollen	Peak Relative Abundance in Pollen	Date of Peak Abundance in Pollen
*T. repens* White Clover	Introduced Perennial Herb	5/15*−9/27	5/15-9/19	53.9%	Multiple peaks
*E. annuus* Daisy Fleabane	Native Annual/ Biennial Herb	5/15*−9/27	5/15-7/24	4.3%	Multiple peaks
*L. japonica* Japanese Honeysuckle	Introduced Perennial Vine	5/15*−9/27	5/15-7/31	14.5%	6/5 GDD 357
*P. lanceolota* Narrowleaf Plantain	Introduced Perennial Herb	5/15*−9/19	5/15-9/4	36.4%	7/17 GDD 896
*R. multiflora* Multiflora Rose	Introduced Perennial Shrub	5/14*−6/14	5/22-5/31	19.1%	5/31 GDD 295
*R. pseudoacacia* Black Locust	Introduced Perennial Tree	5/14*−6/1	5/22-5/31	19.1%	5/31 GDD 295
*P. serotina* Black Cherry	Native Perennial Tree	5/14*−5/22	5/15-5/22	16.8	5/15 GDD 379
*L. vulgare* Wild Privet	Introduced Perennial Shrub	5/18-6/19	6/5-6/19	40.2%	6/19 GDD 493
*G. triacanthos* Honey Locust	Native Perennial Tree	5/23-6/13	5/31	24.8%	5/31 GDD 295
*C. kentukea* Kentucky Yellowwood	Native Perennial Tree	5/24-6/1	Not found in pollen	Not found in pollen	Not found in pollen
*C. intybus* Chicory	Introduced Perennial Herb	5/31-9/20	7/24-7/31	0.4%	7/28 GDD 1056
*R. typhina* Staghorn Sumac	Native Perennial Shrub	6/5-7/4	6/5-6/28	9.1%	6/5 GDD 357
*S. reticulata* Japanese Lilac	Introduced Perennial Tree	6/5-6/25~	6/28	1.9%	6/28 GDD 1078
*S. varia* Crown Vetch	Introduced Perennial Herb	6/6-9/27	Not found in pollen	Not found in pollen	Not found in pollen
*T. americana* Basswood	Native Perennial Tree	6/6-7/10	6/5-6/28	0.4%	6/13 GDD 431
*V. vinifera* Wild Grape	Introduced Perennial Vine	6/6-6/21	6/5-6/19	7.9%	6/5 GDD 357
*Carduoideae spp.* Thistle	Introduced Perennial/ Biennial Herb	6/9-8/28~	7/31-8/9	0.7%	7/31 GDD 1109
*D. carota* Wild Carrot	Introduced Biennial Herb	6/13-9/27	6/13-9/4	5.3%	Multiple peaks
*Melilotus spp.* Sweet Clover	Introduced Biennial/ Annual Herb	6/13-9/24	6/13-8/9	18.8%	7/5 GDD 706
*L. corniculatus* Bird’s-foot Trefoil	Introduced Perennial Herb	6/14-8/9~	6/13-8/31	41.5%	Multiple peaks
*E. vulgare* Vipers Bugloss	Introduced Biennial/ Short Lived Perennial Herb	6/15-7/7	6/28	2.5%	6/28 GDD 599
*L. salicaria* Purple Loosestrife	Introduced Perennial Herb	6/19-9/27	6/28-8/31	13.2%	8/20 GDD 1384
*L. vulgaris* Yellow Toadflax	Introduced Perennial Herb	6/26-9/27	Not found in pollen	Not found in pollen	Not found in pollen
*P. quinquefolia* Virginia Creeper	Native Perennial Vine	7/4-7/17	7/5-7/10	6.1%	7/5 GDD 706
*S. japonicum* Japanese Pagoda Tree	Introduced Perennial Tree	7/6-9/5	Not found in pollen	Not found in pollen	Not found in pollen
*R. japonica* Japanese Knotweed	Introduced Perennial Herb	7/12-9/27	7/19− 9/4	2.9%	Multiple peaks
*V. agnus-castus* Lilac Chaste Tree	Introduced Perennial Tree/Shrub	7/17-8/14	Not found in pollen	Not found in pollen	Not found in pollen
*P. laurocerasus* Cherry Laurel	Introduced Perennial Shrub	7/31-8/28	7/31-8/28	40.7%	8/5 GDD 1171
*A. altissima* White Snakeroot	Native Perennial Herb	8/21-9/27	Not found in pollen	Not found in pollen	Not found in pollen
*E. serotinum* White Bonesets	Native Perennial Herb	8/21-9/27	Not found in pollen	Not found in pollen	Not found in pollen
*A. artemisiifolia* Common Ragweed	Native Annual Herb	9/4-9/27	Not found in pollen	Not found in pollen	Not found in pollen
*A. vulgaris* Mugwort	Introduced Perennial Herb	9/4-9/24	9/4-9/27	38.2%	9/8 GDD 2975
*S. canadensis* Goldenrod	Native Perennial Herb	9/14-9/27	9/19-9/27	35.4%	9/27 GDD 1832
Taxon-Unattributed A	N/A	N/A	9/12-9/27	35.6%	9/12 GDD 1717
Taxon-Unattributed B	N/A	N/A	9/19-9/27	25.9%	9/25 GDD 1807
Taxon-Unattributed C	N/A	N/A	7/31-9/4	62.2%	8/21 GDD 1405
Taxon-Unattributed D	N/A	N/A	7/24-8/31	39.2%	8/14 GDD 1306
Taxon-Unattributed E	N/A	N/A	8/31-9/4	9.0%	9/2 GDD 1562
Taxon-Unattributed F	N/A	N/A	8/31-9/4	21.8%	9/2 GDD 1562
Taxon-Unattributed G	N/A	N/A	6/13	1.4%	6/13 GDD 432
Taxon-Unattributed H	N/A	N/A	7/10-7/19	0.6%	7/10 GDD 788

* indicates plant species that were in bloom when the study began, so date of first bloom could not be determined.

~ indicates species were identified in pollen collected by bees outside of the field observation date; we assume that the food source plant was still in bloom in small quantities in restricted locations within the foraging range.

GDD is the year-cumulative growing degree day in Celsius determined from CLIMOD2 using a base 10°C.

Of the 25 pollen source species positively identified, 47.8% were herbs, 21.7% were trees, 17.4% were shrubs, and 13.1% were vines. A minority of the pollen source species are known to be native to the region (30.4%). Fourteen species (60.9%) were collected by foragers for relatively short time spans (less than five weeks). The predominant pollen source species based on raw relative abundance of the entire seasonal pollen harvest (13,890 pollen grains) were: *T. repens* (24.7%), *P. lanceolota* (8.0%), *L. corniculatus* (7.5%), *A. vulgaris* (5.0%), *P. laurocerasus* (4.2%), *L. vulgare* (3.4%), *L. salicaria* (2.8%), *R. pseudoacacia* (2.5%), *P. serotina* (2.2%), and *S. canadensis* (1.6%). All other identified pollen source species each constituted 1.0% or less of the total seasonal pollen harvest. Pollen source species that reached high relative abundance within weekly samples (i.e., peak relative abundance; see Table 1) include *T. repens* (53.9%), *L. corniculatus* (41.5%), *P. laurocerasus* (40.7%), *L. vulgare* (40.2%), *A. vulgaris* (38.2%), *P. lanceolota* (36.4%), and *S. canadensis* (35.4%). All other identified pollen source species each appeared in peak relative abundance at 25% or less of the weekly samples. The median count of total pollen grains collected was 250 for ubiquitous sources (8 species), 85 for common sources (17 species), and 23 for uncommon sources (6 species). These differences were not significant (Kruskal-Wallis H test χ2(2) = 2.74, p = 0.254, with a mean rank score of 18.56 for ubiquitous, 16.65 for common, 10.75 for uncommon).

### Seasonal trends in pollen foraging choices

The diversity of pollen sources utilized by foragers, measured by Shannon-Weaver diversity index based on average relative abundances for each week, began the season on 15 May relatively low at 1.19, climbed more or less steadily to a peak of 1.61 on 31 July, and then decreased with high variability before reaching 1.01 by the end the experiment on 27 September (Fig 1C). Both the highest diversity (1.67) and the lowest diversity (0.72) were reached within the period of highly variable diversity between 31 July and 27 September. Richness of utilized pollen sources began the season on 15 May at 5.5 species, reached a high on 31 July at 9.0 species, and declined to 4.8 species by 27 September, with the lowest richness for the season occurring at 3.0 on 31 May (Fig 1B). Abundance of collected pollen, measured as wet mass of entire 24-hour pollen harvest, began and ended the season at low levels (<7.0g) but quickly reached a peak 33.7g on 22 May (Fig 1A). Three distinct harvest weight peaks followed over the course of the season on 28 June, 14 August, and 4 September, with harvest weights approaching very low levels on 5 June, 24 July, and 31 August.

The growth habit of the pollen sources utilized by foragers changed over the course of the season. In May, most pollen was collected from herbs (56.3% of all grains), but much was also collected from trees (34.8% of all grains). During the rest of the season trees were not a major source of pollen (0.4% or less), while herbs predominated the pollen samples reaching 88.0% in July. Throughout the season, a minority of the pollen was collected from shrubs (min = 4.6%; max = 19.7%) and vines (min = 1.0%; max = 5.8%).

### Weekly colony pollen foraging dynamics

Foragers from both colonies utilized only a fraction, usually less than half, of the potential pollen food sources known from the field surveys to be in bloom during any given week (Fig 2). On average, 13.7 different pollen source species were known to be in bloom each week, but an average of 5.6 sources (5.1 and 6.0 for Colonies 1 and 2, respectively) were actually utilized by foragers each week. Expressed as a percentage the average weekly utilization was 42.4% of available known pollen food sources (Colony 1 = 39.9%, Colony 2 = 45.0%). The maximum and minimum percentage utilizations were 78.6% on 15 May and 21.4% on 12 September.

Composition of weekly harvests for both colonies is shown in Fig 3 (minor sources under 10% peak relative abundance are not shown for clarity). Late spring (approximately 15 May to 13 June) harvests were dominated by *T. repens*, *P. serotina*, *R. pseudoacacia*, *G. triacanthos*, and *R. multiflora*. Early summer (approximately 19 June to 24 July) was dominated by *T. repens*, *L. vulgare*, *L. corniculatus*, *P. lanceolota*, and *Melilotus spp*. Late summer (approximately 31 July to 31 August) was dominated by mixed asters (Asteraceae), *L. corniculatus*, and taxon-unattributed species. Early autumn (approximately 4 September to 27 of September) was dominated by *A. vulgaris*, *S. canadensis,* and mixed asters (Asteraceae). The two colonies were similar in the identity of pollen species collected each week, while sometimes differing in exact proportions, and with several exceptions such as the collection of *L. corniculatus* beginning 13 June, *R. multiflora* on 5 June, and *L. salicaria* on 31 August.

Foragers utilized novel pollen sources throughout the season (Fig 4). The average number of novel sources utilized on a weekly basis was 2.3, with monthly averages of 2.8, 3.8, 2.0, 1.4, and 1.5 novel sources per week for May, June, July, August, and September respectively. The proportion of the total pollen harvest that was collected from sources utilized for four weeks or less was on average 33.0% (+/- 6.4). However, this short-term use of sources changed over the course of the season; the 15 May – 19 June average was 43.3% + /- 4.4, the 26 June – 24 July average was 3.6% + /- 1.4, the 31 July – 21 August average was 23.5% + /- 6.0, and the 28 August – 11 September average was 74.0% + /- 13.5. For these calculations, dates after 11 September were excluded because the conclusion of the experiment on September 30 prevented determination of the final collection date.

### Pollen foraging phenology

The seasonal time course of pollen collection for all pollen source species, pooled for both colonies, is shown in Fig 5 (see also Table 1). Six pollen sources were utilized throughout the plants’ entire observed bloom. Six pollen source species known to be honey bee food sources, and upon which honey bee foragers were observed gathering pollen, were not utilized at detectable levels by our focal colonies. Fourteen source species were abandoned before the end of their observed bloom. Abandonment for some species was near the end of the observed bloom (e.g., *P. lanceolota*, *R. typhina*), but for others many weeks before the end of the bloom (e.g., *E. annuus*, *L. japonica*, *C. intybus*, *Melilotus spp.*). Many pollen source species were utilized on or near the timing of their first observed bloom with heaviest collection on or near the initial bloom (e.g., *R. typhina*, *V. vinifera*, *P. quinquefolia*, *P. laurocerasus*). For other species utilized at first bloom the heaviest collection was delayed into the bloom (e.g., *L. corniculatus*, *Melilotus spp.*) or showed period bursts of heavy collection (e.g., *D. carota*, *R. japonica*). For some species not utilized at first observed bloom there was a short delay before being detected in collected pollen (e.g., *L. salicaria*, *R. japonica*, *G. triacanthos*), while for others the delay was many weeks (e.g., *C. intybus*, *Carduoideae spp*., *S. reticulata*). Table 2 summarizes the above utilization patterns.

**Table 2:** Classification of Pollen Food Sources by Utilization Pattern.

Source Type	Source Species*
Early adopted, never abandoned	*R. typhina* *V. vinifera* *L. corniculatus* *P. laurocerasus* *A. vulgaris*
Late adopted**, never abandoned	*L. vulgare* *S. reticulata* *Carduoideae spp*.
Early adopted, abandoned	*T. americana* *D. carota* *Melilotus spp.* *P. quinquefolia* *R. japonica* *L. salicaria* *G. triacanthos*
Late adopted**, abandoned	*C. intybus* *E. vulgare*
Rejected	*C. kentukea* *S. varia* *L. vulgaris* *S. japonicum* *A. altissima* *A. artemisiifolia*

* Sources in bloom at beginning of experiment not included; sources still in bloom at end of experiment included under assumption that foraging season was ending imminently.

** Late adopted sources were not seen in more than one pollen sample after their bloom detected in field.

Pollen collection was highly concentrated for some source species (e.g., *L. vulgare*, *R. multiflora*, *R. pseudoacacia*, *G. triacanthos*), while for other species there was no period of heavy collection (e.g., *C. intybus*, *T. americana, Carduoideae spp*.). Peak relative abundances for most species were observed in the first half of the season but varied greatly in terms of their timing within the observed bloom for each species (Table 1, Fig 5). Peak relative abundance was reached immediately upon the first observed bloom for many short-blooming species (e.g., *R. typhina*, *V. vinifera*, *P. quinquefolia*, *P. laurocerasus*, *A. vulgaris*), but not all (e.g., *S. reticulata*, *E. vulgare*). Many species with long observed bloom periods had no single peak relative abundance, being utilized irregularly throughout their bloom (e.g., *T. repens*, *E. annuus*, *L. corniculatus*, *D. carota*, *R. japonica*).

## Discussion

The pollen foraging of the two honey bee colonies in this urbanized northeastern habitat was diverse, dynamic, and showed evidence of strong preferences for certain plant species. At least 33 plant species were collected in detectable amounts, with an average of 6 species in any single 24-hour colony harvest (Fig 1B, Table 1). No evidence of dearth was observed in the number of available sources (Fig 2), though at several short intervals during the season total harvest mass dipped very low (Fig 1A). Foragers adopted novel pollen source species constantly throughout the season, on average 2.3 new species per week (Fig 4), suggesting effective tracking of ephemeral floral sources intertwined with reliance on a set of abundant perennial herbs such as *T. repens* and *P. lanceolota* (Fig 3,5). Some annual sources were utilized immediately following first bloom and never abandoned until last bloom, suggesting strong preference for these pollens (Fig 5, Table 2). Other annual sources were utilized late into their bloom and then abandoned, or never utilized, suggesting rejection of these sources while preferred sources were available (Fig 5, Table 2). These are patterns that have been speculated upon, but to our knowledge never before documented at high (weekly) resolution over an entire foraging season as achieved here. While it is likely that some early spring pollen foraging activity was not documented here, the low weight of the initial pollen harvest on 14 May (GDD 205) and final pollen harvest on 27 September (GDD 1832) suggest most of the active foraging season was captured by this study.

### Seasonal foraging patterns

Pollen foraging choices over the season (Fig 1A) were surprising in several ways. First, there was not a clear peak of pollen harvest mass, richness, or diversity in the late spring despite the expectation that this would reflect swarming preparations and presumed spring bloom patterns. Pollen harvest mass was similar for each seasonal period (late spring = 18.2g + /- 4.2, early summer = 17.6 + /- 3.7), late summer = 15.6 + /- 3.7). Pollen richness and diversity exhibited relatively low levels in the late spring swarming season (richness = 5.2 + /- 0.55, H = 1.4 + /- 0.07) compared with the early summer post-swarming period (richness = 6.2 + /- 0.46, H = 1.8 + /- 0.07). These departures from expectation could be explained if the focal colonies were too weak to attempt swarming. This was, however, not the case as swarming preparations (queen cups, worker congestion, and lack of empty comb) were observed in both colonies during the late spring. A second unexpected result was that pollen harvest mass dipped to very low levels (approaching zero) at least once during each of the three seasonal periods (Fig 1), suggesting occasional limitation of pollen supply rather than a single prolonged pollen-food dearth. These dips, however, did not correspond with a reduced number of species in bloom (Fig 2) or with obvious drops in brood production (from the hive surveys). Therefore, it may be that pollen reserves within the hive allowed the colonies to exercise foraging preference by slowing pollen foraging activity until preferred food sources bloomed. No prolonged periods of low pollen harvests were observed, suggesting that scarcity of pollen was not a limitation to colony fitness in this habitat during this observed year. A third unexpected result was that the richness and diversity of the pollen harvests remained high for most of the season, only dipping to low levels repeatedly at the end of late summer (Fig 1B, 1C). Together with the consistent availability of pollen source species (Fig 2), this suggests that food diversity was also not a limitation to colony fitness in this habitat during this observed year. One exception is the low richness and diversity of the final three pollen harvests in late September (average richness = 3.4 + /- 0.22, H = 1.1 + /- 0.18), which could constrain the production of brood using stored pollen in the early spring of the following season. It should be remembered that these are conservative estimates of both richness and diversity of the honey bee pollen diet because these calculations do not include the 1,573 pollen grains that we were not able to quantify or attribute to a plant taxon due to unusual morphology and low relative abundances.

Weekly pollen source choices (Fig 3) exhibited patterns generally consistent with the division of the pollen-foraging season into three distinct phases (late spring growth, early summer maintenance, late summer hoarding). There is a clear initial phase of 4–5 weeks where the pollen-harvest composition changes radically every week, followed by a second phase of stasis in the harvest composition for 6–7 weeks (from mid-June through the end of July), followed by a third phase with a more variable harvest composition for the last 8–9 weeks of the season (Fig 3). An argument based on these same data could be made, however, for a four-phase pollen-foraging season with a fourth distinct phase marked by the appearance of *A. vulgaris* and *S. canadensis* in September. It is this fourth phase which in this Northeastern U.S. habitat may most truly correspond with winter survival and spring emergence, as this is the pollen most likely to be remaining in the comb when foraging becomes impossible in October. This would imply that the mid-season maintenance period discussed above may be split into two distinct phases for pollen foraging, or that additional adaptive foraging decisions are made during the third phase such as preparations for late-summer swarming [18,20].

Seasonal pollen foraging patterns were overall remarkably similar between the two colonies, but some differences were observed (Fig 3). For example, while Colony 1 maintained a stable mid-season foraging pattern, Colony 2 exhibited marked and sustained collection of one particular species (*L. corniculatus*) from June 13 to July 31. This difference is of a magnitude too large and consistent to be the result of a sampling artifact. The difference suggests that individual colonies either have different optimal resource use or that chance discovery of food sources plays a meaningful role in pollen foraging. The commonness of the plant species in this case suggests the former, but we cannot rule out an element of randomness when it comes to resource discovery. While the focus on two colonies in this study constrains statistical conclusions about inter-colony variation in pollen foraging, these results suggest an intriguing avenue for future research regarding colony-specific foraging dynamics.

### Pollen-foraging dynamics

The observed incorporation of novel pollen sources on a weekly basis throughout the season (Fig 4) highlights the dynamic nature of pollen foraging behavior in honey bees. With an average of 2.3 new sources utilized weekly across the 20-week study period (Fig 4), the colonies demonstrated remarkable flexibility in meeting their nutritional needs in response to a constantly shifting palette of pollen source species. Patterns of pollen-source adoption and abandonment (Fig 5) show that at the colony level honey bees are able to track existing sources, weigh their continued use against new sources that come into bloom, and allocate foraging effort to preferred sources. Source preference might be inferred in relative terms from Table 2 as follows: early adopted never abandoned sources indicate very high preference, late adopted never abandoned sources indicate high preference, early adopted then abandoned sources indicate medium preference, late adopted and then abandoned sources indicate low preference, and entirely rejected sources indicate no preference (i.e., distaste). One example of a dynamic foraging decision was the collection of *A. vulgaris*, a highly preferred source early adopted and never abandoned, coinciding with the abandonment or significant reduction in collection of four other less-preferred source species (*R. japonica*, *L. salicaria*, *D. carota*, *P. lanceolota*). Similarly, the collection of *L. corniculatus* coincided with the abandonment of three less-preferred source species (*D. carota*, *V. vinifera*, *R. typhina*). Abandonment of less-preferred sources also coincides with the adoption of *P. laurocerasus*, *V. vinifera*, and *R. typhina* (Fig 5).

It is possible, though we believe unlikely, that the observed adoptions, abandonments, and switches between floral food sources arise not from colony preferences but simply as an outcome of plant densities and distributions. Formal quantification of floral abundance and density of each pollen source plant in the field was not practical for the present study due to the heterogenous makeup of the foraging area and the predominance of private property. However, qualitative assessment of plant source commonness during field observations corroborates the expectation that the commonness of food-source plants influenced the abundances of pollen collected. For example, the most abundantly collected sources were from ubiquitous plant sources; *T. repens*, *P. lanceolota*, and *L. corniculatus* together accounted for 59.5% of all pollen grains counted in this study. This fits with findings of other studies such as [7] and [44], where a small number of populous long-blooming plants contributed the majority of a colony’s protein diet. In the present study, ubiquitous plant sources had the highest median counts of total pollen grains over the course of the season. However, this trend was not statistically significant when collected amounts of pollen were compared among ubiquitous, common, and uncommon plants (p = 0.254), suggesting that plant abundance alone does not explain the observed foraging dynamics. Furthermore, there were many ubiquitous or common pollen species collected in only small quantities or rejected entirely. These patterns suggest honey bee colonies are as a group exercising strong preferences for which pollen sources to utilize. Future studies where quantification of plant abundance is possible, even in a more homogenous environment, would help further our knowledge of the influence of plant abundance on honey bee pollen-food choice.

### Factors influencing colony pollen preferences

The nature of the present study does not allow us to determine the causal factors underlying the observed colony pollen-foraging preferences, but some speculation in light of the observed patterns seems warranted. Honey bee colonies could maximize their fitness by selectively collecting pollen of high nutritional value, but controversy has been noted [45] about how individual foragers might assess pollen nutritional content given that they do not consume the pollen that they gather. Together with this apparent conceptual contradiction, empirical support for individual workers’ ability to assess the nutritional quality of pollen has been mixed. Some studies show discrimination by workers based on crude protein percentage [46], essential amino acid content [47], or essential fatty acid content [48]. Others have found no evidence for pollen preferences based on nutrition [7,21,41,49,50]. The colony level pollen-foraging preferences observed in the present study might be explained in three non-mutually exclusive ways. First, it may be that we have yet to discover the precise nutritional variable (or combination of variables) that underlies individual pollen preferences as well as the sensory mechanisms that allow pollen foragers to assess that variable in the field. Second, assessment of nutritional value may be indirect, based on signals and cues from other task groups, similar to the regulation of pollen-foraging effort via cues from nurse bees and brood [17,27–29,51,52]. Third, it is possible that honey bee colonies show strong preferences for certain pollen-source plants that are based on characteristics of the plants other than the nutritional value of the pollen, such as the scent strength of the flowers, visual appeal of the flowers’ petals, and/or density of pollen grains per flower (consistent with the results of [49]). A combination of these species-specific floral characteristics might lead a pollen forager to advertise via waggle dances more vigorously for preferred pollen-food patches (consistent with the results of [53]), effectively translating individual-level stimuli into colony level pollen-foraging preferences.

### Phenological calendar of the honey bee-angiosperm mutualism

The data presented here (see Fig 5) illustrate a specific account of overlap between the phenology of angiosperm bloom timing and generalist pollinator behavior for an urbanized habitat in the Northeastern United States. The phenology of 67 species of wild bees was found to be less sensitive to climate variation than was flower phenology [54], suggesting phenological mismatch will be an increasing problem for bees. Phenological mismatch appears to be especially impactful on specialist pollinator bees [55], while impacts on generalist wild bees have been shown to be highly species specific [56]. While the present study of a single season does not allow a direct test of phenological mismatch in honey bees, these data present a phenological calendar for honey bee pollinator-plant interactions that may provide for future comparisons under changing climate or land-use conditions. Phenological calendars that take into account local heat accumulation are used to predict pest-insect development [57] and are being employed to understand how climate change affects pollinator-plant interactions [58]. Grow-degree days (“GDD”) [59] are included in Fig 5 to allow application of the phenological calendar to different locales and years. Under stable climactic conditions, the collection by honey bee colonies of preferred pollen species is expected to follow a regular pattern each year. Climate disruptions that alter food-plant bloom timing threaten to cause phenological mismatch between honey bee and pollen source plants with possible negative fitness outcomes for both. Phenological mismatch could be assessed by comparing the present data set to a future data set and looking for significant differences in the timing of collection of particular pollen species by honey bee colonies. Especially useful as phenological benchmarks are highly preferred short-blooming species such as *R. pseudoacacia* and *G. triacanthos* (peak abundance at GDD 295), *R. typhina* (GDD 357), *L. vulgare* (GDD 493), *P. quinquefolia* (GDD 706), *P. laurocerasus* (GDD 1171), *A. vulgaris* (GDD 1653), and *S. canadensis* (GDD 1832). Mismatch would be especially impactful on honey bee colony fitness if it occurred with pollen source species that composed a large portion of the colony’s diet and bloomed early in the season during peak brood production. Examples from our study include *P. serotina*, *R. multiflora*, *R. pseudoacacia*, and *G. triacanthos* (Fig 3). The continued bloom and abundance of perennial pollen sources such as *T. repens* in our study may mitigate the impacts of phenological mismatch on honey bee colonies. On the other hand, low pollen-food diversity could also negatively impact honey bee health as shown by the experiments of [14]. Long-term monitoring of phenological matching in regions around the globe is essential for understanding and mitigating the impacts of environmental changes on pollinator-plant mutualisms and should be considered a research priority.

## Supporting information

S1 Table Raw Pollen Count Data. The number of pollen grains counted for each taxon over the duration of the study.
(XLSX)
